# A simple approach of three-isocenter IMRT planning for craniospinal irradiation

**DOI:** 10.1186/1748-717X-8-217

**Published:** 2013-09-17

**Authors:** Zheng Wang, Wei Jiang, Yuanming Feng, Yang Guo, Zheng Cong, Bin Song, Yu Guo

**Affiliations:** 1 Department of Radiation Oncology, Huanhu Hospital, Tianjin, China; 2 Department of Radiation Oncology, Tianjin Medical University Cancer Hospital, Tianjin, China; 3 Department of Biomedical Engineering, Tianjin University, Tianjin, China

**Keywords:** Craniospinal irradiation, Field junction, Field edge matching, Overlap-junction IMRT

## Abstract

**Purpose:**

To develop a new IMRT technique to simplify the process and improve efficiency in radiotherapy treatment planning for craniospinal irradiation (CSI) treatment.

**Methods:**

Image data of 9 patients who received CSI treatment in 2012 were used, the prescription was 36Gy in 20 fractions. Two treatment plans were created for each patient, one was with the new technique called three-isocenter overlap-junction (TIOJ) IMRT and the other was with the three-isocenter jagged-junction (TIJJ) IMRT technique. The comparative study was conducted using the parameters of heterogeneity index (HI), conformity index (CI), and doses to the organs at risk (OARs).

**Results:**

Comparing the TIOJ IMRT plans with the TIJJ IMRT plans, the average homogeneity index is 0.071 ± 0.003 and 0.077 ± 0.002, respectively, and the averaged conformity number is 0.80 ± 0.012 and 0.80 ± 0.009, respectively. There are no significant differences (p > 0.05). Both plans provide satisfactory sparing for the OARs.

**Conclusions:**

The TIOJ IMRT technique for CSI treatment planning can create similar plans as with the TIJJ IMRT technique, but the new technique greatly simplifies the steps required to manually set field widths and boundaries and improved efficiency.

## Introduction

Craniospinal irradiation (CSI) has become an important treatment method for primary tumors. Commonly treated tumors include medulloblastoma, high-risk germ-cell tumors, and some radio-sensitive secondary malignant tumors of the meninges. Emerging radiotherapy techniques, such as three-dimensional conformal radiotherapy (3DCRT) and intensity modulated radiation therapy (IMRT), have gradually replaced the traditional large field radiotherapy technology used in CSI treatment. CSI involves complex anatomical structures and requires complex treatment planning, which often entails setting multiple isocenters and matching a large number of fields to obtain satisfactory plans. IMRT technology can offer better comformity Index (CI) and homogeneity index (HI) than traditional multi-field 3DCRT in complex target areas. Inverse treatment planning with IMRT reduces the difficulty of planning and implementation as well. These two advantages are particularly important in CSI. Helical tomotherapy [1,2] and radiotherapy techniques based on protons [3] have also been used in CSI. Another emerging radiotherapy technique, volumetric modulated arc therapy (VMAT), has also been applied in CSI treatment [4,5].

In comparison to other IMRT techniques [6-8], the three-isocenter jagged-junction (TIJJ) IMRT recently proposed by Cao et al. [9] achieves similar CI and HI and simplifies planning and implementation processes. Yet the treatment plan still involves adjustment of a large number of staggered fields, and the planning process is time consuming. Reducing the complexity of treatment plans and shortening treatment time will make the treatment more reliable and improve the overall treatment quality. For this purpose, we have developed a simplified IMRT technique called three-isocenter overlap-junction (TIOJ) IMRT, and presented in this manuscript. The goal is to simplify the implementation of the treatment plan, ensure satisfactory CI and HI, and reduce the time needed for planning and implementing CSI treatment.

## Material and methods

Image data of nine patients who received CSI treatment in 2012 were used for this study (Table 1). Two CSI treatment plans were made for each patient, one was TIOJ IMRT as defined in this manuscript and the other was TIJJ as defined by Cao et al. [9]. The two plans were compared using the parameters of HI, CI, and doses to the organs at risk (OARs).

**Table 1:** Patient demographics

**Patient**	**Gender**	**Age (y)**	**Diagnosis**	**PTV length (cm)**
1	M	8	GCT	62.3
2	M	35	MM	78.6
3	M	13	GCT	64.7
4	M	14	Ependymoma	67.9
5	M	26	GCT	70.8
6	F	30	MM	76.2
7	F	14	GCT	62.5
8	M	10	GCT	68.3
9	F	24	MM	75.6

Patient demographics at time of CT scan acquisition.

Abbrevations: *PTV* Planning target volume, *M* male, *F* female, *GCT* Germ cell tumor, *MM* Medublastoma.

Among the nine patients evaluated, three were average-risk medulloblastoma patients who received reduced dose (23.4 Gy in 13 fractions) while the others received 36 Gy in 20 fractions. To make the plans comparable, we created the plans with the same prescription of 36 Gy in 20 fractions for all patients. The study using patient data was approved by the Ethics Committee of Tianjin Huanhu Hospital (#2012-3).

### Patient position and simulation

All patients were set up in a supine position with both hands naturally and comfortably placed at their sides. A thermoplastic facial mask and a body mask were used to fix the patient. After the immobilization, Six BrainLAB (BrainLAB AG, Feldkirchen, Germany) real-time infrared reflective marker balls were placed on the surface of the thermoplastic masks. The simulation CT images were acquired using a Brilliance CT Big Bore (Philips Medical Systems, Cleveland, OH, USA). Scan range was from the top of the head to the proximal femur, which included the entire torso and arms. Slice thickness was 3 mm.

### Planning

Target delineation: Delineation of planning target volume (PTV) and OARs were both based on CT images. PTV_cns_ included the brain (PTV _brain_) and spinal cord (PTV_spinal_). PTV _brain_ included the whole brain, the meninges and 3 mm beyond their external boundary. PTV_spinal_ included C1 through S3, and 5 mm beyond their external boundary. Lens, optic nerves, eyes, thyroid, heart, lungs, liver, and kidneys were delineated as OARs for comparison.

IMRT plans for all patients were generated using Eclipse treatment planning system (Eclipse TPS 10.0.24, Varian Medical Systems Inc., Palo Alto, CA, USA). A 6MV (Varian 6EX) linear accelerator equipped with a 120-leaf multileaf collimator (MLC) was used to implement the treatment plan. The BrainLAB ExacTrac was used for setup before treatment, moving the table for different isocenters and monitoring the mobility of the patient during treatment.

### TIOJ IMRT plan

**Figure 1:**
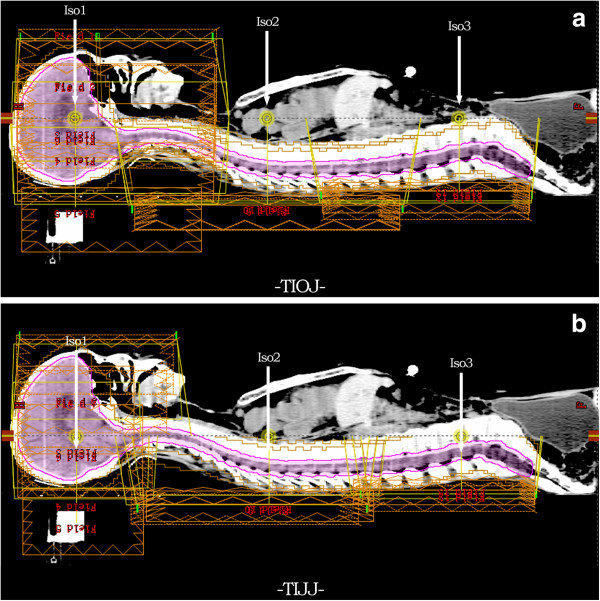
**Beam arrangements for TIOJ plan and TIJJ plan.** Sagittal view shows beam arrangements for both cranial and spinal regions for TIOJ plan **(a)** and TIJJ plan **(b)**. The conventional plan figure shows the placement of isocenters in a TIOJ plan has beams with shorter source to surface distances (SSDs) which make the distances between the MLC and the spinal cord shorter.

The three isocenters of the beams were placed in the TIOJ plan such that they had the same distance of 100 cm to the source, and were denoted as A, B, C, respectively. As shown in Figure 1a, the line connecting the three isocenters (A, B and C) was parallel to and above the midline of the patient in the sagittal plane. All of the PTV_spinal_ were located dorsal to this connecting line. The positions of A, B and C on this connecting line were determined by the following steps. Point A was set as the midpoint along the rostral-caudal direction of the PTV_brain_ in the sagittal plane. If the length of the PTV_cns_ was less than 80 cm, B and C were set to make A, B, and C 25 cm apart to each other. If the length of the PTV_cns_ was greater than 80 cm, B and C were set to make A, B, and C 30 cm apart to each other. This was to ensure sufficient beam overlap among the fields with different isocenters. The collimator angle was set at 0° for all the field sets. The fields in every field set of an isocenter had the same field size. With this set-up scheme, two beam overlap regions with lengths of 15 cm or 10 cm were formed between the three isocenters. The field set with isocenter A (IsoA) contained seven fields, and the gantry angles were 0°, 65°, 100°, 123°, 230°, 257° and 290°, respectively. The field sets with IsoB and IsoC had three radiation fields each, and the gantry angles were 145°, 180° and 215°, respectively. As the distance from spine to treatment machine head for these posterior and posterior oblique fields in the field sets for spinal cord treatment is shorter than 100 cm, the leaf width of MLC projected in the spinal cord region is smaller than its nominal width, this could theoretically provide better homogenous dose distribution in the volumes of field junctions.

The constrains set for the inverse optimizations were as following, 99% of the PTV (for both PTV _brain_ and PTV_spinal_) is to receive 95% of the prescribed dose, the doses to the lens and eyes are to be kept to the lowest achievable, and there are no dose constrains for other OARs.

### TIJJ IMRT plan

The method first proposed by Cao et al. [9] is shown in Figure 1b. Plans covered the cranial and spinal PTVs with the use of three isocenters. Care was taken to ensure that no beam entered through the patient’s shoulders. One isocenter (Iso1) was placed in the cranial PTV, and two isocenters (Iso2 and Iso3) were placed in the spinal PTV, with Iso2 located superior to Iso3. The three isocenters were collinear, and were placed near the patient’s midline. For the patients scanned in the supine position, the Iso1 field set consisted of seven fields with gantry angles of 0°, 65°, 100°, 123°, 230°, 257°, and 290°. Both field sets from Iso2 and Iso3 consisted of three beams with gantry angles of 145°, 180°, and 215°. The collimator angles were set at 0° for all fields. Adjacent field sets were intentionally overlapped to treat a common region of the spine. Field edges were staggered in 1.1 cm steps. The lateral or nearly lateral field cannot treat through the shoulders.

### Plan evaluation

The two plans were compared, mainly using HI and CI of the target areas. Currently there are multiple definitions for HI and CI. The definitions used by Cao et al. [9] were adopted in order to compare the two methods. HI is defined as

(1) $$\mathrm{HI}=\frac{D_{2\%}-D_{98\%}}{D_{\mathrm{Median}}}$$

where D_Median,_ D_2%_ and D_98%_ are doses received by 50%, 2% and 98% the PTV volume, respectively. CI is defined as

(2) $$\mathrm{CN}=\frac{V_{T}{}_{\mathrm{pres}}}{\mathrm{VT}}\times \frac{V_{T}{}_{\mathrm{pres}}}{V_{\mathrm{pres}}}=\frac{V_{T}^{2}{}_{\mathrm{pres}}}{\mathrm{VT}\times V_{\mathrm{pres}}}$$

where V_T pres_ is the targe volume covered by 95% isodose surface. V_pres_ is the treated volume covered by 95% isodose surface and VT is the volume of target. The value of CI is in the range of 0–1, where a value closer to 1 indicates better conformity. The value of HI is in the range of 1–0, where a value closer to 0 indicates better homogeneity.

### Plan quality assurance for the cranial-spinal and spinal-spinal junctions

In complex plans, multiple isocentes can overlap. Homogeneity of dose distribution in overlapping radiation fields of different isocenters must be achieved. The influence of variation in treatment table positioning on dose distribution must be carefully evaluated. In this study, the plan quality assurance (QA) was conducted with an IBA matrix (IBA Dosimetry Germany) The QA plans for the IBA matrix were generated in Eclipse TPS, and the measurements of dose distributions in the overlapped areas of field junctions were performed afterwards. Due to limitations in the size of the IBA matrix test model (27 cm × 27 cm), only dose distribution and pass rate in the overlapping areas were measured in each plan.

### Statistical analysis

Statistical analysis was performed using the SPSS statistical analysis software package, Version 18.0 (SPSS Inc., Chicago, IL). A nonparametric related-samples Wilcoxon signed-ranks test was chosen because the sample sizes were small and not of a normalized distribution, P values <0.05 were considered statistically significant.

## Results

**Figure 2:**
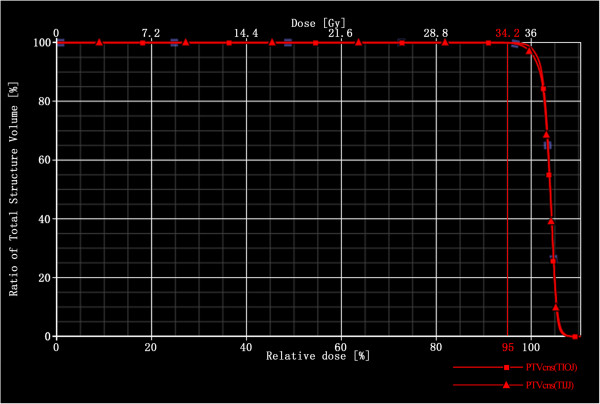
**Dose volume histogram (DVH) comparison of target coverage.** DVH of a representative plan comparing target coverage between the TIOJ plan and the TIJJ plan. The target coverage of both plans meet the initial goal for the target volume coverage.

For the nine patients included in this study, both TIOJ and TIJJ IMRT reach the goal of the 95% isodose curve covering at least 99% of the PTV. Figure 2 shows the PTVcns coverage for patient 5 using a dose volume histogram (DVH). Both plans meet the initial goal for the target volume coverage.

PTVcns coverage, HI, CI and other results in the two plans are listed in Table 2. HI and CI obtained with TIOJ are 0.071 ± 0.003 (Mean ± variance) and 0.80 ± 0.012, respectively. HI and CI obtained with TIJJ are 0.077 ± 0.002 and 0.80 ± 0.009, respectively. Both results meet the PTV dose coverage requirements as published in the International Commission on Radiation Units (ICRU) guidelines [10,11]. There are no significant differences for CI and HI (P > 0.05).

**Table 2:** Evaluation parameters for TIOJ and TIJJ plans

**Patient**	**HI**		**CI**	
	**TIOJ**	**TIJJ**	**TIOJ**	**TIJJ**
1	0.083	0.086	0.83	0.83
2	0.072	0.072	0.74	0.78
3	0.070	0.070	0.81	0.80
4	0.083	0.085	0.86	0.84
5	0.061	0.063	0.80	0.78
6	0.070	0.070	0.79	0.79
7	0.061	0.060	0.81	0.81
8	0.070	0.070	0.83	0.72
9	0.069	0.081	0.76	0.69
Mean ± variance	0.071 ± 0.003	0.077 ± 0.002	0.80 ± 0.012	0.80 ± 0.009

Abbrevations: *CI* conformity index, *HI* homogeneity index.

**Figure 3:**
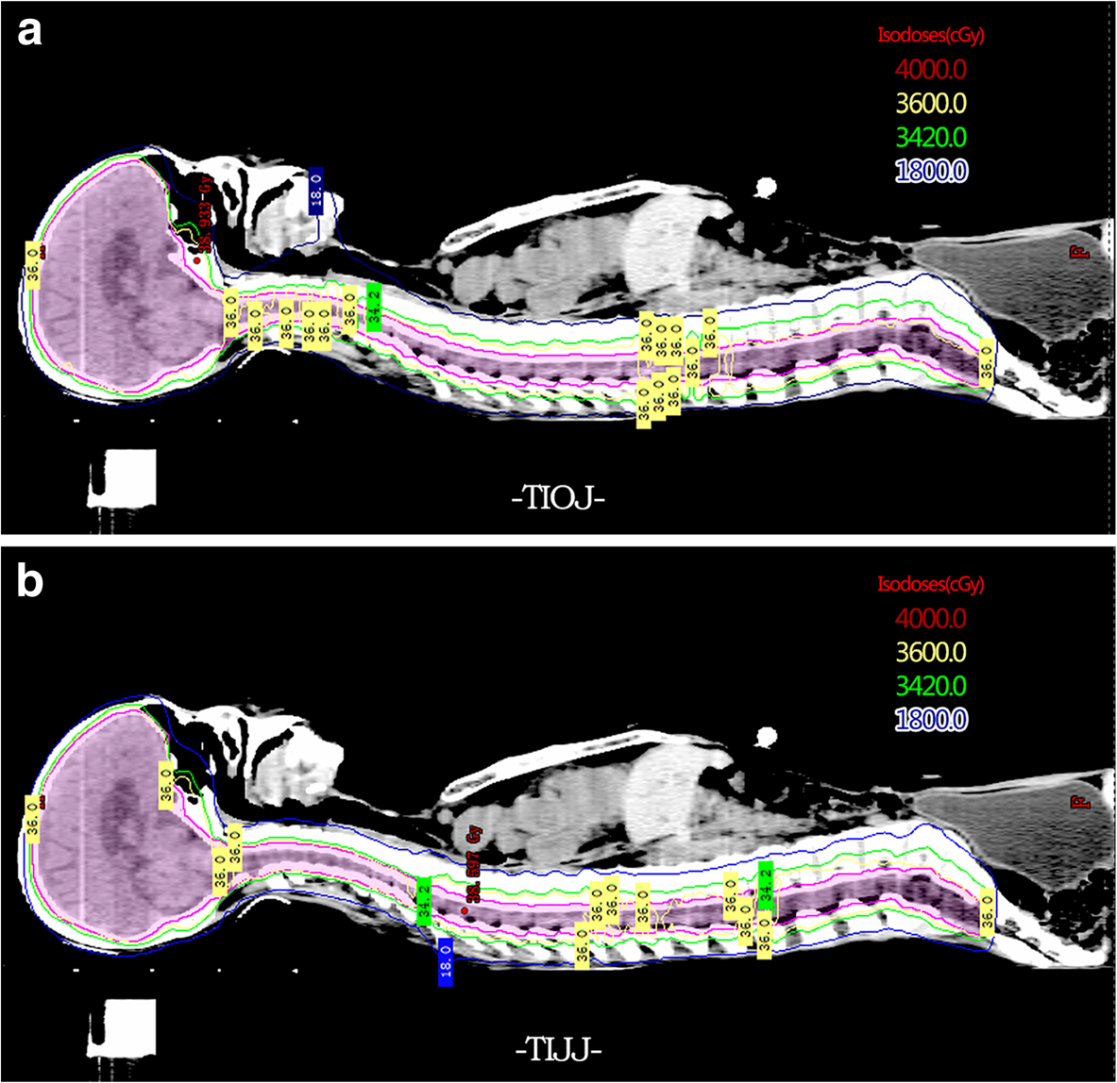
**Isodose distribution of Patient 5 for TIOJ plan and TIJJ plan.** Isodose distribution on a midline sagittal CT slice of Patient 5 for TIOJ plan **(a)** and TIJJ plan **(b)**. Color legend indicates isodose lines, in centigrays. Planning target volume is shown in pink.

Figure 3a and b shows the dose distribution in the two plans in patient 5. No “cold” dosing spots or “hot” dosing spots are found in the radiation beam overlapping regions between isocenters. The two treatment planning techniques provide similar plans in this regard.

The total monitor units (MUs) needed for delivering the fraction dose of 1.8 Gy are 1907.4 ± 60.5 (ranging from 1575 to 2104) with TIOJ plan and 1903.3 ± 34.8 (ranging from 1575 to 2253) with TIJJ plan, respectively, there is no significant difference (P > 0.05). The average doses to the torso in the two plans are 8.75 ± 0.59 Gy with TIOJ and 8.26 ± 0.53 Gy with TIJJ, respectively, there is no significant difference (P > 0.05). No correlation between total MU and torso dose in either plan was found.

**Figure 4:**
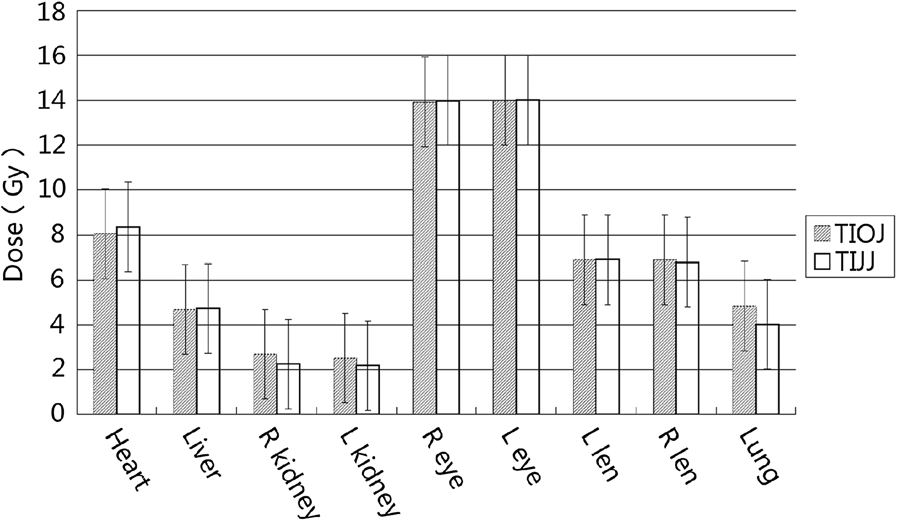
**Median and variation.** Median and variation (shown as bars) of mean doses to OARs in TIOG (shade) and TIJJ (white) plans. L: Left; R: right.

Figure 4 shows the doses to different OARs in the two plans. There are no significant differences in the doses of different OARs with the two plans (P > 0.05).

**Figure 5:**
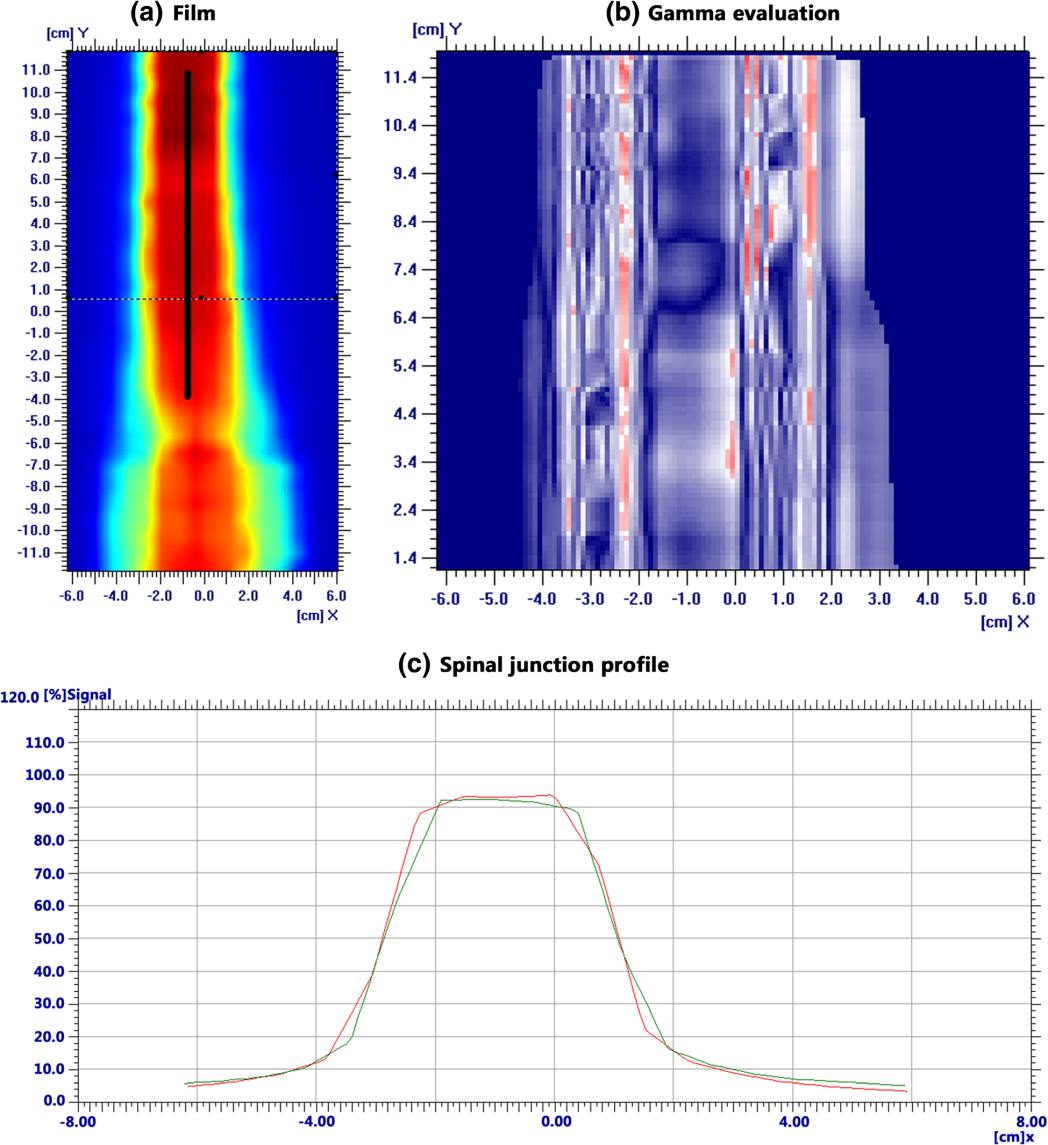
**QA results in the overlapping spinal cord region with TIOJ plan for patient 5. (a)** and **(c)** show the coronal plane dose distributions and profiles for the spinal-spinal junction. The black lines indicate the junction regions. **(b)** shows the gamma index (3% for absolute dose and 3 mm) for the relative dose evaluation.

The comparison of the dose distribution in spinal-spinal beam overlapping regions using an IBA matrix for patient 5 is shown in Figure 5. QA evaluations of TIOJ plans using Gamma index (3% for absolute dose and 3 mm for relative dose evaluation) were performed. All plans has a pass rate above 90%. The mean pass rate is 94.6% (ranging from 92.5% to 97.7%). The measured and planned dose profiles agree well and there are no cold or hot spots.

## Discussion

Simulation, planning, QA and treatment delivery processes for CSI techniques require great care. Patient position, patient immobilization, target delineation, protection of OARs, HI, and the field junctions can have serious effects on the treatment outcome. Compared to traditional 2D radiation therapies, 3D radiation technologies such as IMRT and VMAT [4,5] provide much better target coverage and protection of OARs. Although there have been previous reports of successful applications of IMRT technology in CSI [6,7,12,13], most of these only partially modified or optimized the traditional CSI plan by adding an IMRT component. The technical advantages of IMRT were not fully utilized. Recently, Cao et al. [9] simplified the radiation field matching issue involved in traditional planning by using a single IMRT plan with three isocenters. In practice, this technique requires large amounts of manual adjustment of radiation fields to generate beam overlapping regions which is time consuming.

The TIOJ IMRT technique presented in this manuscript is similar to the ones proposed by Seppala et al. [6] and Cao et al. [9]. But with TIOJ, there is no need for manual adjustment of radiation fields to generate beam overlapping regions and the difficulties in the planning process is greatly reduced while maintaining similar and satisfactory plan results.

As compared to the plan by Cao et al., the placement of isocenters in a TIOJ plan has beams with shorter source to surface distances (SSDs) which make the distances between the MLC and the spinal cord shorter. This makes the projected MLC width smaller in the spinal cord, which is equivalent to having thinner MLC leafs, which helps maximize the potential of the inverse treatment planning system and avoiding hot or cold spots in beam overlapping areas. And with the new TIOJ technique, the lengths of all radiation fields have the maximum length set by MLC (40 cm). In TIJJ, field lengths are manually set. In contrast, larger beam overlap regions can be achieved in the target area with TIOJ, and the planning process involves far less manual setting and adjustment which facilitates dose optimization in the inverse planning system and helps avoid dose cold spots and hot spots in beam overlap regions.

In the TIJJ plan proposed by Cao et al. [9], the filed length in the overlap region between isocenters needs to be manually adjusted in order to generate jagged junction areas with 1.1 cm intervals. During this process, the field width in left-right direction also has to be manually adjusted to avoid excessive radiation field width relative to patient lateral size and MLC leakage. The planning process is complex and time consuming. The proposed TIOJ method only requires three isocenters to be manually determined. The field size and shape are then automatically set by the planning system. The new method greatly simplifies the planning process and reduces planning time. The average time needed to make a TIOJ IMRT plan is about 10 min shorter than that using TIJJ (30 min vs. 40 min). This simplification can also facilitate a standardized design procedure, which minimizes human errors in the process.

VMAT, another emerging radiation therapy technology, has also been applied in CSI treatment [4,5]. With its high dose rate and technical advantages similar to helical tomotherapy, VMAT can satisfactorily solve some CSI related problems (such as a long target area and matching between radiation fields). Lee et al. [5] applied VMAT technology to treat five patients. PTV lengths were 48.1-83.7 cm. The prescription dose was 23.4Gy in13 fractions. The final mean CI and HI were 1.22 (range: 1.09-1.45) and 1.04 (range: 1.03-1.07), respectively. The formulas Lee et al. used to calculate HI and CI were different from ours. For comparison purposes, we used their formulas to re-process the data collected from our nine patients. The CI and HI found with the TIOJ method were 1.23 (range: 1.17-1.34), and 1.08 (range: 1.07-1.11), respectively. Although we could not perform statistical analyses to compare their data with ours, it can be seen that the CI and HI obtained using TIOJ for CSI were very similar to that obtained by Lee et al. using the VMAT technique.

Most patients that receive CSI treatment are teenagers. Before treatment, evaluation of OAR dose needs to be performed to minimize the incidence of radiotherapy-related complications. In the current study, dose limitation requirements were defined for the lens and eyes, similar to the studies by Cao et al. [9] and Lee et al. [5]. Figure 4 shows the OAR doses received with each of the two plans, OAR doses were at a satisfactory level and basically the same with TIOJ and TIJJ. The mean dose of left lens and right lens was 6.89 and 6.91Gy, respectively, using TIOJ. The mean dose of left lens and right lens was 6.91 and 6.78GY, respectively, using TIJJ. These doses were significantly lower than the 9.1Gy, as reported by Cao et al. [9], and significantly lower than the 20Gy and 18Gy reported by Lee et al. [5] with VMAT. These differences may be due to the more stringent limit conditions we set for the lens and eye.

The total MU and the mean dose received by the torso are the most important risk factors for secondary tumors [14-16]. We found that the total MU and dose in the torso using TIOJ were similar to that found using TIJJ. Total MU using TIOJ was 1907.4 ± 60.6 (range: 1575–2104). Total MU using TIJJ was 1903.3 ± 34.8 (range: 1575–2253). So when use the same dose rate, the deliver (beam on) time for both technique (TIOJ and TIJJ) will be very similar. We also found that the total MU were substantially higher than that reported by Lee et al. with VMAT. This suggests that, VMAT technology may be more advantageous than IMRT in reducing the incidence of secondary tumors. We believe that as IMRT treatment involves a large amount of MLC occlusion, total MU may not fully reflect dose received by the whole body. In the current study, mean dose received by the torso was calculated as 8.75 ± 0.59 with TIOJ and 8.26 ± 0.53 with TIJJ. There was no significant difference between the two plans. As Lee et al. did not calculate the torso dose with VMAT, we were not able to make relevant comparisons.

## Conclusions

The TIOJ IMRT method for CSI treatment outlined in this article can creat plans with satisfactory CI and HI. The use of three isocenters and beam overlap regions between the isocenters helps avoid typical CSI problems, such as over-long radiation fields and matching between the fields. As compared to previously reported methods, TIOJ greatly simplified the steps required to manually set field widths and boundaries and improved efficiency. Only one treatment plan and a simple bed capable of moving in one direction are needed to complete the entire treatment. TIOJ IMRT provides a simple and efficient choice for CSI treatment.

## Abbreviations

CSI: Craniospinal irradiation; TIOJ: Three-isocenter overlap-junction; TIJJ: Three-isocenter jagged-junction; HI: Heterogeneity index; CI: Conformity index; OARs: Organs at risk; 3DCRT: Three-dimensional conformal radiotherapy; IMRT: Intensity modulated radiation therapy; VMAT: Volumetric modulated arc therapy; PTV: Planning target volume; MLC: Multileaf collimator; SAD: Source to axis; DVH: Dose volume histogram; ICRU: International commission on radiation units; MUs: Monitor units; SSD: Source to surface distances.

## Competing interests

The authors declare that they have no competing interests.
